# Translation and cross-cultural adaptation of the Hypertension Knowledge-Level Scale for use in Brazil^*^

**DOI:** 10.1590/1518-8345.2832.3073

**Published:** 2018-11-14

**Authors:** Juliana Perez Arthur, Maria de Fátima Mantovani, Maria Isabel Raimondo Ferraz, Ângela Taís Mattei, Luciana Puchalski Kalinke, Roselene de Campos Corpolato

**Affiliations:** 1 Università Cattolica del Sacro Cuore, Facoltà di Medicina e Chirurgia, Brescia, BS, Italy.; 2 Universidade Federal do Paraná, Departamento de Enfermagem, Curitiba, PR, Brazil.; 3 Universidade Estadual do Centro Oeste, Departamento de Enfermagem, Guarapuava, PR, Brazil.; 4 Hospital do Trabalhador, Unidade de Terapia Intensiva, Curitiba, PR, Brazil.

**Keywords:** Knowledge, Hypertension, Surveys and Questionnaires, Validation Studies, Nursing, Health Education

## Abstract

**Objective:**

to make the translation, cross-cultural adaption and content and face validation of the Hypertension Knowledge-Level Scale for use in Brazil.

**Methods:**

methodological research carried out in six stages: translation, synthesis, back-translation, expert committee’s assessment, pre-test and validation. Validation was performed through the Delphi technique in two rounds. The participants were two translators and two back-translators, eight professionals in the expert committee, 40 adult participants in the pre-test, 35 experts in the first validation round and 28 in the second validation round. Data analysis included Cronbach’s alpha, content validity index and one-tailed t-test.

**Results:**

the translation and cross-cultural adaptation allowed for language adjustments so that the items were comprehensible and suitable for use in Brazil. The content validity index of the Brazilian version of Hypertension Knowledge-Level Scale was 0.96 and Cronbach’s alpha was 0.92.

**Conclusions:**

the scale was translated, cross-culturally adapted to Brazilian Portuguese, had its content and face validated and proved reliable to evaluate the knowledge of adults about hypertension.

## Introduction

Knowledge involves the acquisition of information and skills through teaching and experiences^1^. Its measurement in the health area is important because it can help professionals to plan care actions and educational activities.

When it comes to chronic diseases such as systemic arterial hypertension (SAH), the measurement of knowledge about the disease is a factor that affects adherence to therapy^2^. Because SAH is a silent and aggressive disease, its treatment depends on the knowledge, collaboration and active participation of the patient. It is known that informed people are more likely to positively change the way they care for their health^3^ ^-^ ^4^. Moreover, lack of knowledge and incorrect beliefs regarding hypertension are factors that influence and limit the quality of life^2^.

In this sense, Turkish researchers developed the Hypertension Knowledge-Level Scale (HK-LS) to evaluate the knowledge about SAH of adults over 18 years of age. The original version of this instrument was published in English in 2012^5^ and has been used in many parts of the world.

The HK-LS was used in Iran in 2015 to determine relevant factors to knowledge on hypertension, including treatment and control^6^. In 2016, it was translated and cross-culturally adapted to the Arabic language and used to assess the knowledge of Jordanian adults about SAH^1^. The instrument was also adapted to the Greek language^7^ and, in Poland, a study was published reporting its use in the Polish version^8^.

Translation and cross-cultural adaptation can be useful when an instrument was created by researchers in a different country or reality; in order to ensure the quality of a new version of such instrument, well-designed scientific procedures are necessary^9^. Currently, this process is already used in several areas of study and stands out for having benefits over the creation of new instruments, such as reduced cost and time and the possibility of comparing different realities^10^.

The translation and cross-cultural adaptation of the HK-LS to the Brazilian reality makes it possible to provide a valid and reliable instrument already used in other realities for the measurement of knowledge of adults about hypertension. Therefore, the objective of this research was to translate, cross-culturally adapt, and validate the content and face of the Hypertension Knowledge-Level Scale for use in Brazil.

## Method

This is a methodological research of translation, cross-cultural adaptation and content and face validation of the HK-LS carried out from April 2016 to October 2017.

The HK-LS consists of 22 statements divided into six sub-dimensions: definition (items 1 and 2); medical treatment (items 6, 7, 8 and 9); drug compliance (items 3, 4, 5 and 12); lifestyle (items 10, 11, 13, 16 and 17); diet (items 14 and 15); and complications (items 18, 19, 20, 21 and 22)^5^. Each statement was designed to be answered in the Likert-type scale format with three options: correct, incorrect or don’t know. The maximum scale score is 22, being scored only when the respondent provides the right answer^5^.

The process of translation and cross-cultural adaptation was based on international guidelines^11^ ^)^ and consists of six stages: 1 - Initial translation; 2 - Synthesis of translations; 3 - Back-translation; 4 - Eexpert committee; 5 - Pre-test; 6 - Face and contente validation.

In initial stage (1), the instrument was translated by two independent bilingual translators from the original language, English, into the target language, Brazilian Portuguese. One of the translators was from the health area, the other had no knowledge in the area; each one produced an independent version, called T1 and T2.

In the stage 2, the translations were synthetized through a consensus meeting between the two translators and two nursnig researchers who work with SAH. In this meeting, the necessary reformulations were made, giving rise to the synthesis version of the initial translations, which was called T12.

In the stage 3, the synthesis version T12 was back-translated from Portuguese to English by two other bilingual independent translators who were unaware of the original instrument. Each back-translator produced a new version, called BT1 and BT2, and this stage aimed to evaluate if the content of the synthesis version was similar to the original instrument.

After feedback, an expert committee (stage 4) made up of eight professionals: two translators, two back-translators, two nursing PhD university professors, and two postgraduate (stricto sensu) nursing students with experience in the theme studied. The experts met with the purpose of producing the pre-final version of the instrument and did the analysis of the semantic, idiomatic and conceptual equivalence of the translated version.

In stage 5, the version produced in the previous stage was subjected to a pre-test. This stage was carried out in a health unit in the city of Curitiba, Paraná. Forty (40) individuals were invited and accepted to participate, as recommended by the methodological framework used^11^. They met the following inclusion criteria: adult aged 18 to 60 years; duly registered in the health unit where the pre-test was carried out; literate person. The exclusion criterion was: presence of any limiting factor that could prevent the reading and filling of the questionnaire.

Participants responded to the instrument and then evaluated it for intelligibility, appearance, clarity, and wording. They were also encouraged to give suggestions for improvements where they considered it appropriate. For the comparison between groups, a one-tailed t-test was applied and the level of significance adopted was p < 0.05.

The stage 6 consisted in the, content and face validation through the online Delphi technique, with help of Brazilian researchers who are experts in the theme studied. The Content Validity Index (CVI) was used to evaluate the agreement between researchers, and it was established that the rounds of content validation would be carried out until a CVI ≥ 0.8 was obtained. This value was based on a bibliographic review, which found values 0.5 and 0.8^12^ to indicate consensus levels among the specialists for this type of evaluation; however, it is known that the higher the CVI, the better is the agreement among the experts. The Cronbach’s alpha was calculated to evaluate the reliability.

The platform of the National Council of Scientific and Technological Development was consulted for recruitment of experts. A search was performed with the key words and Boolean operators: “Systemic arterial hypertension” AND “Nursing” AND “Chronic disease”, resulting in a total of 241 experts.

After recruitment, an invitation was sent to the experts to participate in the validation process that contained the results of the previous steps together with a questionnaire for content and face validation of the instrument.

**Figure 1:**
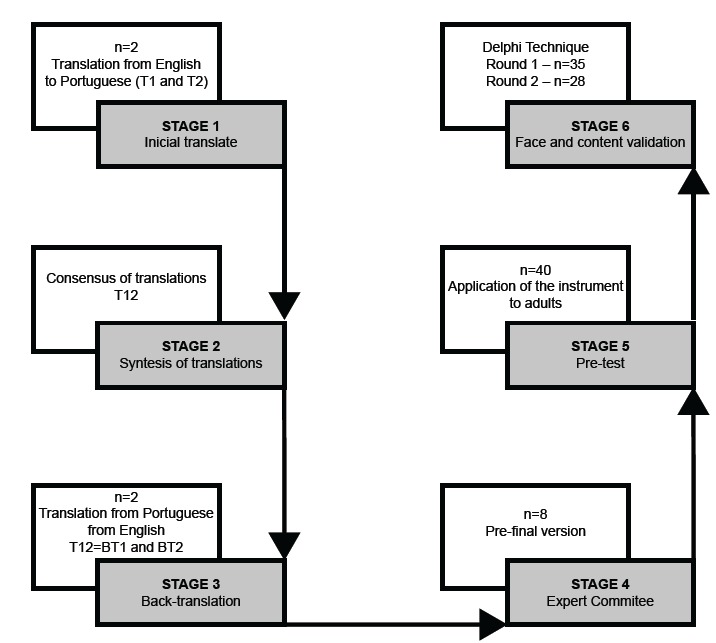
Flowchart of the stages of cross-cultural adaptation and face and content validation of the HK-LS* for use in Brazil. Curitiba, PR, Brazil, 2018

Two rounds of content validation were required to reach a CVI ≥ 0.8. The participants were able to evaluate the instrument translated in a quantitative way, using a Likert-type scale of 1 to 4 points and also qualitatively by offering suggestions of changes. The instrument was evaluated for clarity, applicability, face and content of each statement. Figure 1 shows the flowchart of all stages of the research.

* HK-LS - Hypertension Knowledge-Level Scale

The research was approved by the Research Ethics Committee of the Federal University of Paraná under Opinion number 1,689,333 and authorized by the Ethics Committee of the Municipal Health Secretariat of Curitiba under protocol 104/2016. The study followed the principles of resolution 466/2012.

## Results

In the stage 1, the most frequent discrepancies found in the translations were related to words or terms with similar meanings in Brazil (e.g.: increased blood pressure and high blood pressure; medications and medicine; meals and foods; salt and salty foods).

In stage 2, the synthesis of translations, all the discrepancies found in the previous stage were studied and the professionals chose the term they considered more usual in Brazilian Portuguese. At this stage, we also identified the need to change the past tense to the present tense.

In addition, the sentence number 11 of the instrument was subjected to cross-cultural adaptation and the term “boiling or grilling”, initially translated as “boiling or grilling” was changed to “cooking only in water or grilling” because it was considered that the initial translation used a term that is not usual in the Brazilian context.

In the stage 3 the back-translated versions BT1 and BT2 were identical in eight (66.7%) statements and all the differences of back-translations were considered synonyms. In this way, we concluded that the T12 version corresponded to the original instrument. In the stage 4, the expert committee made relevant changes, improvements and cross-cultural adaptations in order to produce the version that was used in the pre-test (stage 5).

As to sociodemographic characteristics of the participants of the pre-test, 28 (70%) were female; 19 (47.5%) were aged between 19 and 39 years and 21 (52.5%) between 40 and 60 years; 29 (72.5%) were married or common-law married; 20 (50%) had up to nine years of schooling and 20 (50%) had nine to 16 years of schooling. Regarding occupation, 21 (52.5%) were employed and 26 (65%) reported not having a diagnosis of SAH.

Regarding the responses of the instrument, the average total percentage of correct answers was 74.7%. The sub-dimension with the lowest percentage of correct answers was “definition”, with average of 46.2%; and the one with the highest percentage of correct answers was the “lifestyle”, with average of 89.5%.

The participants of the pre-test were separated into two groups: with and without a diagnosis of hypertension. The mean percentages of correct answers of people with hypertension were higher in all sub-dimensions of the instrument, as Table 1.

**Table 1:** Average number of correct answers (n = 40) of the groups of participants with and without hypertension. Curitiba, PR, Brazil, 2018

Subdimension	Overall average (%)	Average people with SAH* (%)	Average people without SAH* (%)
	(n=40)	(n=14)	(n=26)
Definition	46.2	60.7	38.5
Medical treatment	66.9	71.7	64.4
Drug compliance	81.9	82.1	81.7
Lifestyle	89.5	92.9	87.7
Diet	75	92.9	65.4
Complications	71.5	88.6	62.3
p-value^†^		0.0035	

*SAH - systemic arterial hypertension; †p-value - One-tailed t-test

In the stage 6, face and content validation, experts from all regions of Brazil participated in the two rounds of validation. In round 1 the general evaluations for Cronbach’s alpha and CVI were 0.92 and 0.84, respectively.

Eight sentences obtained CVI below 0.80 in round 1, namely: sentence 1, 2, 4, 5, 6, 20, 21 and 22. Regarding the comments of the experts, the following stood out: need to adapt technical terms to the lay public; adapt words to the Brazilian context; and clarify dubious questions.

After round 1, the items that obtained the CVI below 0.80, and those to which pertinent suggestions were given, were reformulated. After the changes, a feedback was prepared and sent to the participants, who were invited to participate in the next round. In round 2, the Cronbach’s alpha score remained 0.92 and the CVI increased to 0.96. In this round all the evaluated questions obtained the CVI above 0.80; therefore, the validation process was ended. The CVIs of each item evaluated in each of the rounds are presented in Table 2.

**Table 2:** Content Validity Indices obtained in rounds 1 and 2 of the validation process per evaluated item. Curitiba, Brazil, 2018

Evaluated item	CVI* Round 1	CVI* Round 2
Appearance	0.94	1
Clarity	0.80	0.96
Applicability	0.91	1
Item 1	0.66	0.86
Item 2	0.77	0.89
Item 3	0.86	†
Item. 4	0.77	0.93
Item. 5	0.74	1
Item. 6	0.54	1
Item. 7	0.91	†
Item. 8	0.91	†
Item. 9	0.91	†
Item. 10	0.86	†
Item. 11	0.94	†
Item. 12	0.91	1
Item. 13	0.91	1
Item. 14	0.89	†
Item. 15	0.91	†
Item. 16	0.97	†
Item. 17	0.83	1
Item. 18	0.97	0.93
Item. 19	0.89	0.96
Item. 20	0.71	0.96
Item. 21	0.74	0.96
Item. 22	0.71	0.96

*CVI - Content Validity Index; † - Item not evaluated in round 2.

**Figure 2:**
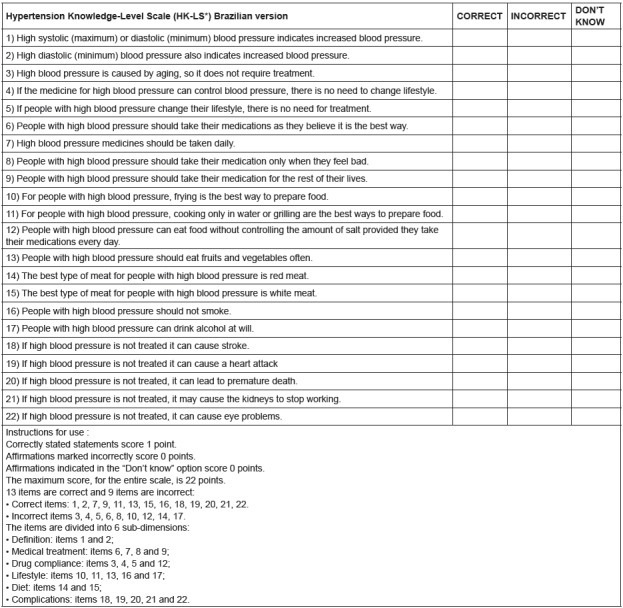
Brazilian version of the HK-LS * and usage guidelines. Curitiba, PR, Brazil, 2018

Figure 2 shows the translated and adapted version of the HK-LS for use in Brazil and the guidelines for its use.

* HK-LS - Hypertension Knowledge-Level Scale

## Discussion

During the process of translation and cross-cultural adaptation of the scale, grammatical and cultural adjustments were necessary in order to adapt it to the Brazilian context. The methodology used for this process indicates that the researcher, with the purpose of ensuring the comprehension and the correct choice of words of the translated version, must guarantee that the instrument is clearly understood by the target population^11^.

Questions about cultural relevance can appear at any point in the translation process. It is necessary to discuss the nature of the problem in order to find alternatives that will not significantly alter the meaning of the phrase ^13^. Thus, all the stages of this research sought to adjust the instrument to the Brazilian adult population and the efforts were directed so that the version was adequate to be applied to people from diverse sociocultural contexts.

The application of the pre-test (stage 5) allowed a critical view of the instrument with respect to the answers obtained. The sub-dimensions with the highest and the lowest number of correct answers were “lifestyle” (89.5%) and “definition” (46.2%), respectively. In a study that applied the Arabic version of the HK-LS, the sub-dimension “lifestyle” was also the one with the highest average number of correct answers (84.22%); however, the lowest average was found in the sub-dimension “diet” (52.11 %) followed by “definition” (58.09%)^1^.

A significant difference (p = 0.0035) was found between respondents of the groups “with SAH” and “without SAH” in the pre-test, and those with the disease obtained higher percentages of correct answers. This difference was also found in a study that used the Arabic version of the HK-LS to evaluate the knowledge of Jordanian adults^1^.

Considering that knowledge about the disease affects the way people practice self-care, adhere to therapy and control related risk factors^6^, the high number of correct answers in the group with SAH are desirable. It is assumed that when patients have the disease, they know more about it, and they are better prepared for taking care of themselves. On the other hand, it is important that the general population, i.e. people not affected by SAH but with related risk factors, be informed about what is hypertension and what forms of prevention and treatment are available.

To promote knowledge about SAH in the population, one possible use of the HK-LS is in preventive and educational actions in primary health care. In this sense, the translated and adapted scale can be applied to assess the prior knowledge of these people, so that, before this evaluation, individual or collective needs can be listed and health improvement actions undertaken with the objective of avoiding or delaying the onset of the disease or its complications.

The information collected through the measurement of knowledge can be used in the elaboration of educational activities. Nurses have a fundamental role in this context as they are in constant dialogue with patients and are able to promote social and individual transformations that favor the improvement of self-care and/or reduce damages before illnesses^14^.

Regarding the content and face validation of the scale, the participation of experts from all regions in Brazil was important due to the continental proportions of the country and regional and cultural differences.

Regarding reliability, the original version of the HK-LS in English obtained a Cronbach’s alpha of 0.82^5^; in the Brazilian version this value was 0.92. In turn, the study that translated and adapted the scale to Greek evaluated reliability in two groups, with and without SAH, and obtained a reliability of 0.66 and 0.79, respectively^7^. Although the values ​​found were unequal, they all indicated that the scale is reliable to be applied in the realities for which they are intended.

It was not identified a need to change the number of items in the scale or the evaluation form of the scale. Thus, the Brazilian version of the HK-LS is also composed of 22 items separated into six sub-dimensions, with a maximum score of 22 and minimum of zero.

As limitations of the research, we emphasized that the scale has gone through the process of adaptation and validation in few countries, making it difficult to discuss and compare the results.

## Conclusions

The HK-LS was translated, cross-culturally adapted to Brazilian Portuguese, had its content and face validated, and proved to be reliable to evaluate the knowledge of adults about hypertension. The adoption of steps according to the chosen methodological framework was important to guarantee the quality of the final result.

Considering that the cross-cultural adaptation of the HK-LS in the Brazilian version obtained good indices of validation and reliability. The instrument can be used to evaluate the knowledge about SAH in several areas of health, such as primary care, to plan care and health education activities, as well as to evaluate the general population.

From a teaching perspective, the HK-LS may also be useful for the purpose of encouraging students to use valid and reliable instruments to assist them in their professional practice
